# Low hemoglobin, albumin, lymphocyte, and platelet score increases symptomatic intracranial hemorrhage risk in thrombectomy patients

**DOI:** 10.3389/fneur.2025.1588875

**Published:** 2025-08-04

**Authors:** Shuaiyu Chen, Zhihang Huang, Jingwen Qi, Bin Wei, Yan E, Chunmei Liu, Yingdong Zhang, Xiaohao Zhang

**Affiliations:** Department of Neurology, Nanjing First Hospital, Nanjing Medical University, Nanjing, Jiangsu, China

**Keywords:** HALP index, large vascular occlusive stroke, symptomatic intracranial hemorrhage, mechanical thrombectomy, predictor

## Abstract

**Background:**

The HALP index, a composite biomarker integrating hemoglobin, albumin, lymphocyte, and platelet parameters, reflects both immunological competence and nutritional status. We therefore conducted this retrospective analysis to assess the correlation between HALP score and the risk symptomatic intracranial hemorrhage (sICH) risk in patients receiving mechanical thrombectomy (MT).

**Methods:**

This retrospective study included patients with acute ischemic stroke due to anterior circulation large vessel occlusion who underwent MT between October 2019 and July 2024. The HALP index was calculated based on admission laboratory parameters. The sICH was diagnosed according to Heidelberg Bleeding Classification criteria within 72 h post-procedure. Multivariate logistic regression analysis was performed to identify independent association between pretreatment HALP score and sICH risk after adjusting for covariates.

**Results:**

A total of 735 patients (mean age, 71.3 ± 10.9 years; 423 male) were enrolled in this study. sICH was diagnosed in 82 patients (11.2%) during hospitalization. After adjusting for demographic characteristics and other covariates, multivariate logistic regression analysis revealed that a low HALP index was significantly associated with an elevated risk of sICH (odds ratio: 1.058, 95% confidence interval: 1.024–1.094, *p* = 0.001). findings were obtained when the HALP score was analyzed as a categorical variable. Additionally, the restricted cubic spline analysis confirmed a linear inverse relationship between the HALP index and the risk of sICH following MT (*p* = 0.551 for non-linearity).

**Conclusion:**

Our data confirmed a significant inverse correlation between the HALP score and the sICH risk in patients treated with MT. This suggests that the HALP score may serve as a valuable tool for monitoring and managing sICH risk in ischemic stroke patients following reperfusion therapy.

## 1 Introduction

Ischemic stroke is a leading cause of disability and mortality worldwide, imposing substantial medical and financial burdens globally (1, 2). Numerous randomized controlled trials have established that mechanical thrombectomy (MT) is effective for ischemic stroke patients with large vessel occlusion in the anterior circulation (3). Symptomatic intracranial hemorrhage (sICH) is a serious complication following reperfusion therapy, strongly associated with neurological deterioration and poor functional outcomes (4, 5). Therefore, early and accurate prediction of sICH is essential for optimizing functional recovery after MT.

Stroke outcomes are influenced by a multitude of factors, including genetic predisposition, modifiable lifestyle choices, dietary patterns, and cardiometabolic conditions. Inflammatory processes play a significant role in these mechanisms. Extensive research has shown that lymphocytes can alleviate neuronal damage by regulating inflammatory responses and act as key immunomodulators during acute ischemic stroke (6). Beyond their role in thrombosis, platelets also contribute to the inflammatory response triggered by ischemic stroke, primarily through interactions between platelet receptors and endothelial or immune cells (7). The Thrombolysis in Stroke Patients registry, which included 7,533 stroke patients treated with intravenous thrombolysis, demonstrated that a lower platelet count was significantly associated with an increased risk of sICH, while a higher platelet count correlated with a greater risk of mortality (8). Additionally, malnutrition is linked to stroke-related complications. Previous studies have indicated that low hemoglobin levels can lead to cerebral hypoxia, mitochondrial dysfunction, and neuronal damage (9). Deficiencies in serum albumin have been associated with poorer 90-day outcomes in patients with large artery occlusive stroke following MT (10), partly due to reduced antioxidant capacity after ischemic injury. Recently, the HALP (hemoglobin, albumin, lymphocyte, and platelet) index has emerged as a validated marker for evaluating systemic inflammation and nutritional status (11). The HALP index has gained recognition as a novel predictor of clinical outcomes in various cancers (12) and has also been shown to predict stroke recurrence and cognitive impairment in ischemic stroke patients (13, 14). However, the relationship between the HALP score and sICH in patients undergoing thrombectomy remains unclear.

Therefore, we conducted this study to evaluate the association between the HALP index and the risk of sICH in ischemic stroke patients following MT.

## 2 Materials and methods

### 2.1 Subjects and study design

We retrospectively analyzed acute ischemic stroke patients who underwent MT at Nanjing First Hospital between October 2019 and July 2024 for large vessel occlusion in the anterior circulation. The inclusion criteria were as follows: (1) age 18 years or older; (2) presence of anterior circulation large vessel occlusion (internal carotid artery or middle cerebral artery M1/M2 segments); and (3) availability of data necessary for calculating the HALP score. To ensure the homogeneity of the study population, we excluded patients with malignant tumors or hematological disorders. All procedures performed in studies involving human participants were reviewed and approved by the Ethics Committee of Nanjing First Hospital. This study complies with the Declaration of Helsinki. Due to its retrospective nature; patient consent was waived by the Ethics Committee of Nanjing First Hospital. Patient data confidentiality was maintained in Nanjing First Hospital.

### 2.2 Baseline variables assessment

We collected data on demographic characteristics, vascular risk factors, imaging findings, procedural details, and laboratory results. The National Institutes of Health Stroke Scale (NIHSS) was used to evaluate baseline neurological deficits (15). The Alberta Stroke Program Early CT Score (ASPECTS) was applied to measure pre-treatment infarct volume (16). Stroke subtypes were classified according to the Trial of Org 10,172 in Acute Stroke Treatment (TOAST) criteria (17). Collateral circulation was evaluated using digital subtraction angiography and graded according to the American Society of Interventional and Therapeutic Neuroradiology/Society of Interventional Radiology (ASITN/SIR) scale, with grades 0–1 indicating poor collateral circulation and grades 2–4 representing moderate to excellent collateral circulation (18). Successful recanalization was defined as a modified Thrombolysis in Cerebral Infarction (mTICI) score of 2b–3 (19).

### 2.3 Measurement of HALP score

Fasting whole blood samples were collected within 24 h of admission and systematically processed for all laboratory analyses. The HALP index was calculated using the following formula: hemoglobin levels (g/L) × albumin levels (g/L) × lymphocyte counts (/L) / platelet counts (/L) (20).

### 2.4 Definition of sICH

sICH was diagnosed based on the Heidelberg Bleeding Classification within 72 h post-MT (21).

### 2.5 Statistical analysis

Quantitative variables were expressed as mean ± standard deviation (SD) or median (interquartile range [IQR]), depending on the distribution normality, while categorical variables were presented as frequencies (percentages). Continuous variables were compared using the Student’s *t*-test or Mann–Whitney U test, and categorical variables were analyzed using the chi-square test or Fisher’s exact test. Binary logistic regression analysis was conducted to assess the association between the HALP score and the likelihood of sICH. Model 1 was adjusted for age and sex; Model 2 was further adjusted for baseline ASPECTS and poor collateral circulation. In model 3, additional adjustments were made for demographic characteristics and variables with a *p* value <0.1 in the univariate analysis, including baseline NIHSS score, baseline ASPECTS, prior intravenous thrombolysis, poor collateral status, fasting blood glucose, hemoglobin, and lymphocyte count. Variance Inflation Factor (VIF) was used for evaluating multicollinearity between HALP and its components. Furthermore, the pattern and magnitude of the relationship between the HALP index and sICH risk were evaluated using restricted cubic splines with three knots (at the 5th, 50th, and 95th percentiles), adjusted for the covariates included in Model 3. We also used the receiver operating characteristic (ROC) curve analysis to detect the discriminative ability of the HALP index in predicting sICH.

All statistical analyses were performed using IBM SPSS Statistics (version 25.0) and R software (version 4.3.1). A two-tailed *p* value <0.05 was considered statistically significant in this study.

## 3 Results

### 3.1 Baseline characteristics

This study enrolled 735 consecutive ischemic stroke patients, with 57.6% being male and a mean age of 71.3 ± 10.9 years. Among them, 265 patients (36.1%) received intravenous thrombolysis prior to endovascular treatment (EVT). Poor collateral circulation was observed in 328 patients (44.6%), and successful reperfusion was achieved in 91.7% of cases. Table 1 provides detailed clinical data stratified by HALP quartiles. Significant differences were observed across HALP quartiles in baseline NIHSS scores, poor collateral circulation, and fasting blood glucose levels. However, demographic characteristics and vascular risk factors showed no significant differences among the groups.

**Table 1:** Baseline characteristics stratified by the quartile of HALP score.

Variables	HALP sore				*P* value
	First quartile, *n* = 184	Second quartile, *n* = 184	Third quartile, *n* = 184	Fourth quartile, *n* = 183	
Demographic characteristics					
Age, years	72.0 ± 10.8	72.7 ± 9.3	70.4 ± 12.0	70.1 ± 11.2	0.158
Male, *n* (%)	104 (56.5)	103 (56.0)	109 (59.2)	107 (58.5)	0.909
Vascular risk factors, *n* (%)					
Hypertension	145 (78.8)	148 (80.4)	146 (79.3)	137 (74.9)	0.590
Diabetes mellitus	81 (44.0)	94 (51.1)	80 (43.5)	70 (38.3)	0.091
Hyperlipidemia	17 (9.2)	22 (12.0)	23 (12.5)	26 (14.2)	0.528
Coronary heart disease	39 (21.2)	25 (13.6)	36 (19.6)	29 (15.8)	0.206
Smoking	82 (44.6)	85 (46.2)	83 (41.5)	76 (41.5)	0.828
Clinical data					
Systolic blood pressure, mmHg	140.8 ± 23.3	139.6 ± 22.4	137.9 ± 22.5	138.9 ± 21.7	0.700
Diastolic blood pressure, mmHg	84.2 ± 14.3	84.3 ± 13.5	84.0 ± 13.5	84.9 ± 14.5	0.918
Time from onset to recanalization, min	368.0 (250.0, 538.0)	355.0 (255.0, 570.0)	344.0 (220.0, 555.0)	375.0 (230.0, 552.5)	0.683
Baseline NIHSS, score	15.0 (11.0, 19.0)	14.0 (10.0, 17.0)	13.0 (10.0, 17.0)	12.0 (9.0, 17.0)	0.003
Baseline ASPECTS, score	9.0 (8.0, 10.0)	9.0 (8.0, 10.0)	9.0 (8.0, 10.0)	9.0 (8.0, 10.0)	0.831
Stroke subtypes, *n* (%)					0.063
Atherosclerotic	68 (37.0)	73 (39.7)	90 (48.9)	91 (49.7)	
Cardioembolic	93 (50.5)	90 (48.9)	68 (37.0)	72 (39.3)	
Others	23 (12.5)	21 (11.4)	26 (14.1)	20 (10.9)	
Prior intravenous thrombolysis, *n* (%)	75 (40.8)	60 (32.6)	64 (34.8)	66 (36.1)	0.416
Poor collateral status, *n* (%)	84 (45.7)	79 (42.9)	77 (41.8)	72 (39.3)	0.020
Successful reperfusion, *n* (%)	170 (92.4)	166 (90.2)	165 (89.7)	173 (94.5)	0.311
Number of stent-retriever passes	1.5 ± 1.2	1.6 ± 1.2	1.5 ± 1.3	1.49 ± 1.07	0.878
sICH, *n* (%)	39 (21.2)	22 (12.0)	15 (8.2)	6 (3.3)	0.001
Vascular occlusion site, *n* (%)					0.943
Middle cerebral artery	122 (66.3)	123 (66.8)	126 (68.5)	126 (68.9)	
Internal carotid artery	62 (33.7)	61 (33.2)	58 (31.5)	57 (31.1)	
Laboratory parameters					
Fasting blood glucose, mmol/l	7.5 ± 2.4	7.2 ± 2.3	7.1 ± 2.6	7.0 ± 2.4	0.014
Hemoglobin, g/L	119.7 ± 20.2	124.2 ± 17.5	133.3 ± 16.0	138.6 ± 16.9	0.001
Leukomonocyte, 10^9/L	0.7 ± 0.3	1.0 ± 0.3	1.2 ± 0.4	1.7 ± 0.7	0.001
Albumin, g/L	35.8 ± 3.6	37.1 ± 3.5	37.8 ± 3.4	38.9 ± 3.4	0.001
Platelet count, 10^9/L	208.7 ± 55.6	193.1 ± 45.2	189.8 ± 51.2	155.4 ± 45.8	0.001

HALP, index combined by hemoglobin, albumin, lymphocyte and platelet; NIHSS, National Institute of Health stroke scale; ASPECTS, the Alberta Stroke Program Early Computed Tomography Score; TOAST, Trial of Org 10,172 in Acute Stroke Treatment; sICH, symptomatic intracranial hemorrhage.

### 3.2 Risk factors of sICH after MT

The mean HALP score among the study participants was 32.2. Based on the Heidelberg Bleeding Classification, 82 patients (11.2%) were diagnosed with sICH. Table 2 summarizes the age, sex, baseline clinical data, and laboratory findings of patients with and without sICH. Univariate analysis revealed that patients with sICH had higher NIHSS scores (median: 15.0 vs. 13.0; *p* = 0.044) and lower baseline ASPECTS scores (median: 8.0 vs. 9.0; *p* = 0.003) compared to those without sICH. Prior intravenous thrombolysis (46.3% vs. 34.8%; *p* = 0.040) and poor collateral circulation (62.2% vs. 42.4%; *p* = 0.001) were more common in patients with sICH than in those without. Additionally, patients with sICH exhibited higher fasting blood glucose levels (mean: 8.2 ± 2.35 mmol/L vs. 7.07 ± 2.41 mmol/L; *p* = 0.001) and lower hemoglobin concentrations (1.1 ± 0.4 g/L vs. 1.2 ± 0.6 g/L; *p* = 0.046) compared to those without sICH. The HALP index was significantly lower in patients with sICH than in those without (mean: 26.2 ± 13.4 vs. 32.8 ± 19.7; *p* = 0.001).

**Table 2:** Clinical characteristics of study participants according to patients with and without sICH.

Variables	With sICH, *n* = 82	Without sICH, *n* = 653	*P* value
Demographic characteristics			
Age, years	72.2 ± 9.9	71.18 ± 11.0	0.405
Male, *n* (%)	50 (61.0)	382 (58.5)	0.668
Vascular risk factors, *n* (%)			
Hypertension	66 (80.5)	497 (76.1)	0.378
Diabetes mellitus	39 (47.6)	286 (44.1)	0.525
Hyperlipidemia	8 (9.1)	80 (90.1)	0.512
Coronary heart disease	17 (14.6)	112 (20.7)	0.422
Smoking	32 (39.0)	253 (38.7)	0.894
Clinical data			
Systolic blood pressure, mmHg	141.8 ± 22.0	139.0 ± 22.5	0.278
Diastolic blood pressure, mmHg	83.3 ± 13.6	84.5 ± 13.9	0.486
Time from onset to recanalization, min	376.5 (253.8, 533.0)	354.5 (240.0, 556.3)	0.746
Baseline NIHSS, score	15.0 (11.0, 19.0)	13.0 (10.0, 18.0)	0.044
Baseline ASPECTS, score	8.0 (8.0, 9.0)	9.0 (8.0, 10.0)	0.003
Stroke subtypes, *n* (%)			0.226
Atherosclerotic	43 (52.4)	290 (44.4)	
Cardioembolic	33 (40.2)	279 (42.7)	
Others	6 (7.3)	84 (12.9)	
Prior intravenous thrombolysis, *n* (%)	38 (46.4)	227 (34.8)	0.040
Poor collateral status, *n* (%)	51 (62.2)	277 (42.4)	0.001
Successful reperfusion, *n* (%)	72 (87.8)	602 (92.2)	0.175
Number of stent-retriever passes	1.7 ± 1.3	1.5 ± 1.2	0.176
Vascular occlusion site, *n* (%)			0.338
Middle cerebral artery	52 (63.4)	445 (68.1)	
Internal carotid artery	30 (36.6)	208 (31.9)	
Laboratory parameters			
Fasting blood glucose, mmol/l	8.2 ± 2.4	7.1 ± 2.4	0.001
Hemoglobin, g/L	124.9 ± 19.4	129.4 ± 19.7	0.046
Lymphocyte, 10^9/L	1.1 ± 0.4	1.2 ± 0.6	0.075
Albumin, g/L	36.7 ± 4.5	37.3 ± 3.5	0.327
Platelet count, 10^9/L	191.3 ± 48.5	186.3 ± 53.9	0.422
HALP score	26.4 ± 13.4	32.8 ± 19.8	0.001

sICH, Symptomatic Intracranial Hemorrhage; mRS, Modified Rankin Scale; NIHSS, National Institute of Health stroke scale; ASPECTS, the Alberta Stroke Program Early Computed Tomography Score; TOAST, Trial of Org 10,172 in Acute Stroke Treatment; HALP, index combined by hemoglobin, albumin, lymphocyte and platelet.

### 3.3 Associations between HALP index and sICH risk

**Figure 1:**
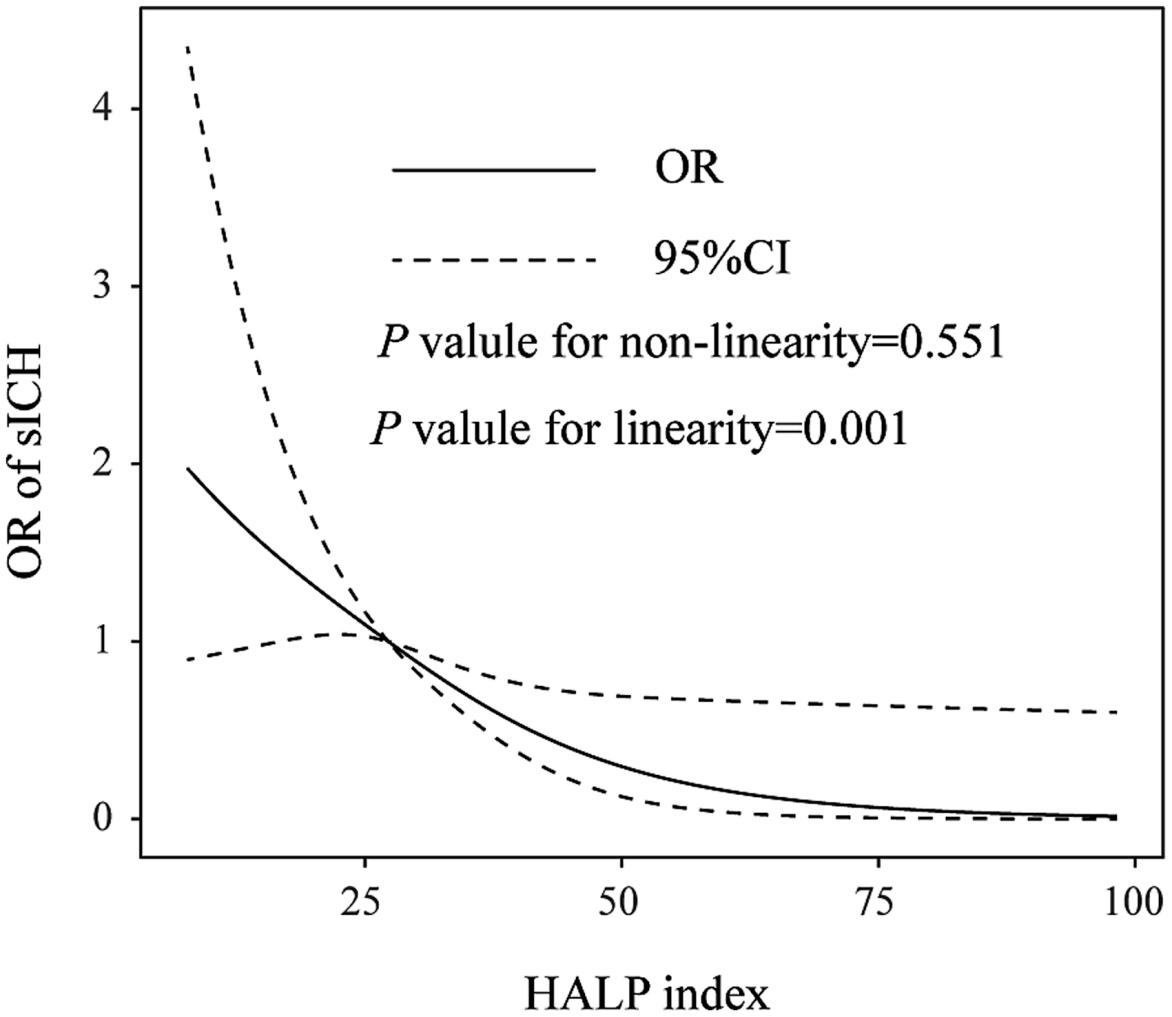
Restricted cubic spline plots depicting the relationships between the HALP score and the risk of sICH. Multivariable adjusted odds ratio (OR, represented by solid lines) and 95% confidence intervals (CI, represented by dotted lines) for the risk of sICH in model 3 are presented. The median value of the HALP score was designated as the reference.

**Figure 2:**
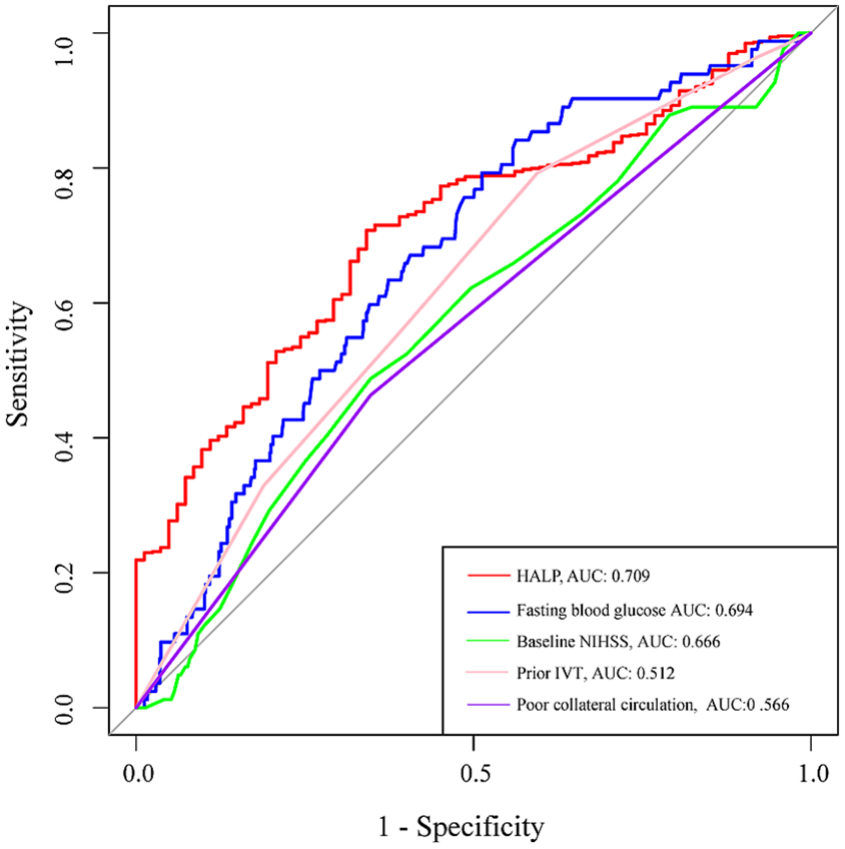
Receiver operating characteristic curve analysis evaluates the predictive value of HALP index and other established predictors for sICH.

After adjusting for covariates, multivariate logistic regression analysis revealed that a higher HALP index was significantly associated with a reduced risk of sICH (odd ratios, 1.058; 95% confidence interval, 1.024–1.094, *p* = 0.001). These findings were further supported when the HALP index was analyzed as a categorical variable (1st quartile vs. 4th quartile of HAPL score, odd ratios, 7.342; 95% confidence interval, 2.962–18.200, *p* = 0.001; Table 3). Additionally, restricted cubic spline analysis confirmed a linear inverse relationship between the HALP index and sICH risk (*p* = 0.551 for non-linearity; *p* = 0.001 for linearity; Figure 1). In the ROC analysis, the area under the ROC curve values of the HALP index were 0.709 (95% CI: 0.657–0.761, *p* = 0.027; Figure 2). Also, the predictive ability of the HALP index is superior to baseline NIHSS score (AUC = 0.666), baseline glucose levels (AUC = 0.694), poor collateral status (AUC = 0.566), and prior IVT treatment (AUC = 0.512).

**Table 3:** Multivariate regression analysis for the association between HALP score and sICH risk.

Variables	Model 1	*P* value	Model 2	*P* value	Model 3	*P* value
	OR (95% CI)		OR (95% CI)		OR (95% CI)	
HALP (per 1-unit decrease)	1.048 (1.027–1.072)	0.001	1.047 (1.025–1.070)	0.001	1.058 (1.024–1.094)	0.001
HALP quartile						
First	7.934 (3.268–19.265)	0.001	8.298 (3.394–20.287)	0.001	7.342 (2.962–18.200)	0.001
Second	4.006 (1.585–10.128)	0.003	3.900 (1.537–9.898)	0.004	3.832 (1.492–9.838)	0.005
Third	2.618 (0.993–6.907)	0.052	2.528 (0.955–6.691)	0.062	2.365 (0.881–6.355)	0.088
Fourth	Reference		Reference		Reference	

HALP, index combined by hemoglobin, albumin, lymphocyte and platelet; sICH, symptomatic intracranial hemorrhage; OR, odds ratio; CI, confidence interval.

Model 1, adjusted for age and gender.

Model 2, adjusted for baseline ASPECTS and poor collateral status.

Model 3, adjusted age, gender and variables with a *P* value < 0.1 in the univariate analysis including baseline NIHSS score, baseline ASPECTS, prior intravenous thrombolysis, poor collateral status and fasting blood glucose.

## 4 Discussion

This study identified the HALP index as an independent predictor of sICH in patients with large artery occlusive stroke undergoing MT. This association remained significant even after adjusting for potential confounders, suggesting that the HALP index could serve as a modifiable risk factor for thrombectomy patients.

The HALP index, calculated from hemoglobin levels, albumin levels, lymphocyte counts, and platelet counts, reflects the interplay between inflammation and nutritional status. Previous clinical studies have demonstrated that a lower HALP index is strongly associated with an increased risk of cardiovascular diseases and overall mortality (22, 23). The HALP index also demonstrates a significant correlation with the risk of post-stroke cognitive impairment (24) and poor outcomes (13). Even though the underlying mechanisms connecting the HALP score and sICH remain unclear, the components of the index might shed light on these observations. Firstly, systemic inflammation is thought to be associated with increased endothelial dysfunction and the impairment of the blood—brain barrier following cerebral ischemia (25). Moreover, highly reactive platelets are indicative of a severe pro-inflammatory state (26, 27). Additionally, platelets can interact with platelet-binding T lymphocytes to produce cytokines, interferons, and chemokines. As a result, this interaction modifies the blood—brain barrier after a stroke (28). Secondly, lymphocytes assume pivotal regulatory functions in the inflammatory process subsequent to ischemic stroke (29). A decrease in lymphocyte levels has been associated with larger infarct volumes and higher NIHSS scores (30). Significantly, both of these factors are independent predictors of sICH (31). Thirdly, hypoalbuminemia contributes to the occurrence of sICH. It does so by intensifying the inflammatory response and oxidative stress (32), stimulating platelet aggregation (33), and enhancing cytokine adhesion within the postcapillary microcirculation (34). Finally, robust evidence demonstrates that reduced hemoglobin levels can give rise to brain hypoxia, mitochondrial dysfunction, and neuronal damage, thus increasing the risk of hemorrhage (9). Additional research is essential to thoroughly clarify the role of the HALP score in predicting the risk of sICH after reperfusion therapy.

We demonstrated that 82 patients (11.2%) experienced sICH during hospitalization. This percentage is slightly higher than the rates reported in several randomized controlled trials (3), as well as those observed in the MR CLEAN Registry (5.8%) (35) and the NASA Registry (9.9%) (36). The data in these registries were predominantly obtained from white populations. This disparity might be attributed to the higher prevalence of cerebral atherosclerosis in the Asian population. Intracranial atherosclerotic occlusion is often more difficult to recanalize successfully compared to cardioembolic occlusion, since the former is frequently associated with significant stenosis. Notably, in this study, a correlation was found between sICH and the number of passes made with the retriever. Repeated use of the thrombectomy device for atherosclerotic occlusion may lead to vascular endothelial damage and disrupt the blood–brain barrier, both of which are linked to an increased risk of sICH (37). However, differences in study methods and the definitions of sICH could potentially explain this discrepancy.

Several limitations in our study need to be acknowledged. First, there is an absence of clinical data on whether patients have pre-existing factors affecting the calculation of the HALP, such as a history of infection and medications. Secondly, given the nature of our study design, this research was unable to establish a causal relationship between HALP and the risk of sICH. Thirdly, the study failed to document whether the patients had been taking medications capable of influencing HALP scores prior to the commencement of the study. Examples of such medications include anti—platelet drugs and iron supplements. Finally, this research was executed at a solitary hospital in China and encompassed a particular cohort of cerebral infarction patients. This fact may potentially restrict the applicability and generalizability of the study findings. Consequently, multicenter studies involving large and diverse sample sizes are imperative to validate and strengthen our research findings.

In summary, this study clearly demonstrated that low HALP index was significantly associated with the risk of sICH in patients suffering from large artery occlusive stroke subsequent to MT therapy. The HALP score holds the potential to enhance the early and precisely-targeted treatment of stroke for these high-risk patients. Further research is essential to evaluate the potential effectiveness of dietary interventions and anti-inflammatory therapies in preventing sICH following MT treatment.

## Data Availability

The original contributions presented in the study are included in the article/supplementary material, further inquiries can be directed to the corresponding authors.
